# HBV/HDV Co-Infection: Epidemiological and Clinical Changes, Recent Knowledge and Future Challenges

**DOI:** 10.3390/life11020169

**Published:** 2021-02-22

**Authors:** Caterina Sagnelli, Evangelista Sagnelli, Antonio Russo, Mariantonietta Pisaturo, Laura Occhiello, Nicola Coppola

**Affiliations:** Department of Mental Health and Public Medicine, University of Campania “Luigi Vanvitelli”, 80131 Naples, Italy; caterina.sagnelli@unicampania.it (C.S.); antonio.russo2@unicampania.it (A.R.); mariantonietta.pisaturo@unicampania.it (M.P.); laura.occhiello@gmail.com (L.O.); nicola.coppola@unicampania.it (N.C.)

**Keywords:** HDV, hepatitis D, epidemiology, pathogenesis, therapeutics

## Abstract

Several investigations have been published on Hepatitis Delta Virus (HDV) infection in recent years, from which we have drawn the salient data to provide readers with useful information to improve their knowledge on the subject. HDV genotypes 5–8 have been recently imported to Western countries from central Africa, whose clinical relevance deserves further investigation. Ongoing HDV replication has been identified as an independent predictor of progression to cirrhosis and HCC for patients with HDV chronic hepatitis (HDV-CH). Long-term treatments of HDV-CH with standard or pegylated interferon alfa (peg-IFN-α) have all been unsatisfactory, leading to a sustained virological response (SVR) only in 20–30% of patients treated, faced with a poor tolerability and frequent serious adverse reactions; the addition of HBV nucleo(s)tide analogues to peg-IFN- α did not improve the rate of SVR. The improved knowledge of the HDV life cycle has allowed the development of direct acting agents towards key-points of the HDV life cycle, namely bulevirtide, lonafarnib and nucleic acid polymers. Preliminary data have shown that these drugs are more effective than interferon-based therapies, but adverse reactions are also common, which however seem toned down in combination therapy with other antivirals.

## 1. Introduction

The need for the narrative review article we propose to readers stems from the extensive literature developed in recent years on different aspects of hepatitis D virus (HDV) infection. The structure and life cycle of the virus have become well known, and this has allowed the production of new agents directly acting on the HDV life cycle. Variations in HDV epidemiology have concerned countries that were already widely studied, linked in part to the intensification of migratory flows from areas at high HDV endemicity to Western countries and in part to the consistent reduction in hepatitis B virus (HBV) circulation following the worldwide application of universal HBV vaccination programs. Furthermore, new knowledge has been acquired regarding geographical areas that were poorly investigated previously.

Many reports have been published in the past on the diseases associated with HDV infection, but some clinical aspects of the more advanced phases of the illness (development of liver failure and hepatocellular carcinoma-HCC) have been more thoroughly investigated. Of great clinical relevance today is the possible diversification in the clinical expression and therapeutic response to the new antiviral agents of HDV genotypes 5–8 imported in the last two decades into Western countries from central Africa, compared to the traditional genotypes 1 and 2 [1,2,3,4,5,6].

The numerous therapeutic attempts made in the past did not provide satisfactory results. The administration of 9 million units of standard interferon-alfa (IFN-α) three times a week for 24–48 weeks provided a sustained virological response (SVR) in only 20–30% of patients treated, little in comparison to the low tolerability and the associated serious adverse reactions; it is of little wonder that this therapy became obsolete. Pegylated interferon alfa (peg-IFN-α) did not substantially improve the efficacy of the cure, although it was better tolerated and less burdened by adverse reactions. In addition, the combination of peg-IFN-α with the nucle(s)tide analogues, developed to inhibit HBV replication, did not improve the rate of SVR obtained with peg-IFN- α alone, a predictable event based on the knowledge of the HBV and HDV life cycle.

New antiviral agents directed against different replicative phases of the HDV life cycle have recently been produced and are in the initial phase of clinical experimentation, with preliminary favorable results. They too are burdened by adverse reactions, but their use in combination with other antiviral drugs seems to tone down these side effects.

The availability of new information on different aspects of HDV infection led us to produce this narrative review, which is aimed particularly at doctors and researchers who carry out their activities, in part or in full, in the field of hepatic diseases.

## 2. Structure and Life Cycle of HDV

**Figure 1:**
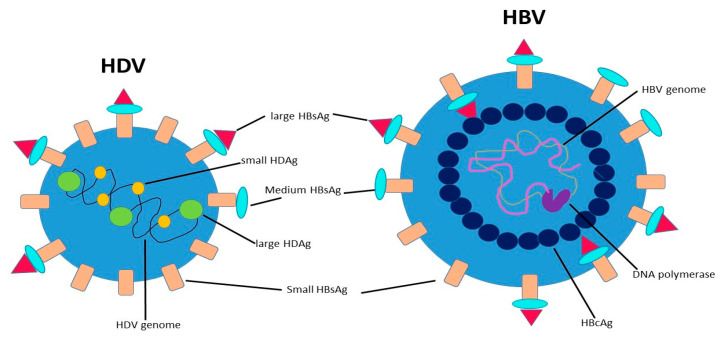
Hepatitis B virus (HBV) shares its small, medium and large hepatitis B surface antigens (HBsAg) with hepatitis D virus (HDV), acting as a helper virus of HDV.

**Figure 2:**
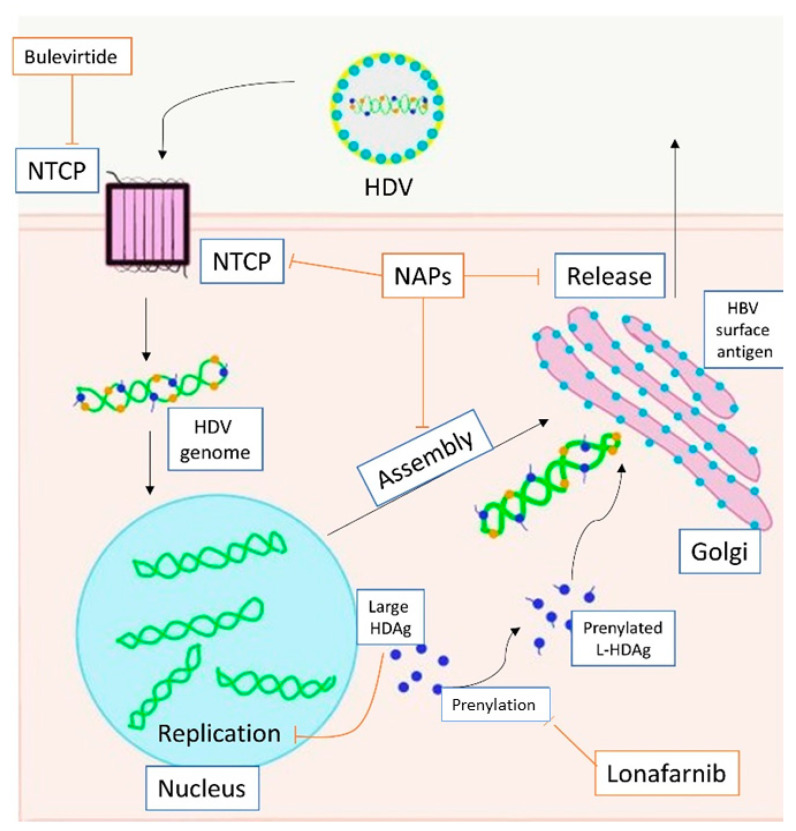
Action of direct anti-HDV agents on the HDV life circle. HDV enters the hepatocyte through NTCP. The replication is carried out in the nucleus. Large HDAg is farnesylated in the cytoplasm. Assembly ends in the Golgi apparatus with the bond between HBsAg and HDAg. Bulevirtide inhibits NTCP (entry), lonafarnib inhibits prenylation (farnesylation, assembly), NAPs inhibit NTCP (entry), farnesylation (assembly) and release. Footnotes: NTCP, sodium taurocholate co-transporting polypeptide; HDV, hepatitis D virus; HDAg, hepatitis D antigen; L-HDAg, large hepatits D antigen; HBV, hepatitis B virus; NAPs, nucleic acid polymers.

HDV is a small RNA virus with a diameter of about 36 nm, with a nucleocapsid and a 1.7Kb circular ss RNA of variable length in relation to genotype [7] (Figure 1). The HDV genome is the smallest viral genome capable of infecting mammals and looks more like viruses that infect plants than those that infect humans [8]. HDV is a defective virus that requires the HBsAg PreS-1 domain of L-HBsAg for its assembly; it lacks its own envelope, and its outer coat consists of components taken from HBV. HDV, however, does not need active HBV replication for its synthesis, because the translation of its structural proteins can also be guaranteed by the HDV RNA that is integrated alone within the hepatocytes. HDV encodes only for the antigenic protein HD-Ag, synthesized in two forms: the small HD-Ag (S-HD-Ag) and the large HD-Ag (L-HD-Ag), which are structurally identical, but L-HD-Ag has an extra 19 aa chain in the C-term [9]. HDV infects the hepatocytes through the myristoylated N-terminus of the pre-S1 domain of the L-HBsAg and its multiple transmembrane receptor sodium taurocholate co-transporting polypeptide (NTCP) [7]. After entry, HDV genome translocates into the nucleus through HD-Ag [8] and uses host RNA polymerase II, a DNA-directed RNA polymerase, to transcript HDV RNA by a rolling-circle mechanism [9] like that of plant viroids (Figure 2) [10]. First, a multimeric linear transcript is synthesized; then, using autocatalytic self-cleaving sequences, ribozymes, this linear transcript is cleaved into monomers [11]. RNA monomers are ligated by cellular RNA ligase into an antigenomic, monomeric and circular RNA used as a template for a rolling-circle replication [12]. Three different RNAs are generated: HDV genome, anti-genome and a smaller anti-genome that contains the open reading frame for HD-Ag [13,14]. S-HD-Ag has a fundamental role in replication itself, as it is required for RNA synthesis, while L-HD-Ag has a role in the packaging. Farnesylation enables the interaction between HD-Ag and HBsAg. At C-terminal for L-HD-Ag, there is a 19-amino acid polypeptide that includes C-terminal CXXX-box motif, a substrate for prenyltransferases, which adds a prenyl lipid group (farnesyl group) [15,16]. When HBsAg is completed, farnesylation anchors the protein to the endoplasmic membrane enabling the interaction through HD-Ag and HBsAg, and this allows the release of virus particles.

## 3. HDV Epidemiology, Unchanged Knowledge and Changes over Time

Nearly 70 million subjects are infected with HDV worldwide [17], with an anti-HDV seroprevalence among HBsAg-positive carriers varying according to the geographical area of birth, socio-economic status and exposure to risk factors [18,19]; a high prevalence of HDV chronic carriers have been observed in Central Africa, South America, Turkey, Mongolia, some Pacific island countries (Kiribati), southern Italy and the previous Soviet Union [18,20,21,22]. A systematic review and meta-analysis reported a worldwide HDV seroprevalence of 14.6% among HBV-positive patients, a percentage much higher than previous estimates of approximately 5%, with the highest seroprevalence in the intravenous drug users (IVDU), in subjects with unsafe sexual behavior and in cohabitants with HBsAg-positive family members [17].

Eight HDV genotypes have been identified, of which 7 present more than 90% similarity over the entire genomic sequence, while HDV-genotype 3 exhibits a 40% divergence with the other 7 at the nucleic acid level; in addition, 2-4 subtypes have been identified so far for each genotype [18,23,24]. HDV-genotype 1, the most represented worldwide, prevails in western Europe and North America [25]; HDV-genotype 2 was previously confined to Asia but it has recently emerged in Egypt and Iran [26,27]. HDV-genotype 3 has been found in North and South America, mostly in the Amazon basin [28], and HDV-genotype 4 has been found in China and Japan [29]. Central Africa is the main site of HDV diversification, with HDV-genotypes 1, 5, 6, 7 and 8 [29].

Several epidemiological surveys have shown an increased prevalence of HDV infection in migration-destination countries where this infection was previously uncommon [30,31,32,33,34,35]. In addition, the geographic distributions of HDV genotypes have changed over time in Western countries, mostly due to the intensification of immigration from endemic countries. In fact, HDV genotypes 5, 6 and 7 have been detected in several European countries [36,37,38]. In Australia, immigrants born in Africa have been found at higher risk of carrying HDV infection than native Australians (RR= 1.55; 95% CI 1.14–2.09) [30].]. In England, more than half the patients with HDV infection have come from continents or sub-continents where HDV infection is endemic (southern or Eastern Europe, Africa, Middle East, Asia) [32]. A similar event has occurred in Greece, where immigrants are more than half of the HDV population [34]. Changes in molecular epidemiology have also been described for HBV infection; in Italy, related to migratory flows, the “traditional” HBV genotype D has been replaced in part by other HBV genotypes in the latter decades, prevalently introduced from Africa and Eastern Europe and responsible for nearly 40% of acute hepatitis B [39,40,41,42,43]. In Italy, there was a substantial decrease in HBV endemicity consequent to changes in socio-economic conditions and to the universal HBV vaccination that started in 1991 and is still ongoing. Consequently, HDV infection among HBsAg positive subjects progressively decreased from the 24% detected in 1981 [44] and the 23.4% found in 1987 [45] to 14.4% in 1992 [46], 8.3% in 1997 [47], 8.1% in 2007 [19] and to 6.4% in 2019 [48], when, reflecting a survival effect, HDV-related chronic hepatitis affected older subjects with advanced disease. Instead, this HDV prevalence has increased in immigrants from 2001 to 2013 (from 12.2% to 26.4%), due to the increase in migratory phenomena from Africa and Eastern countries and the difficulties of vaccinating undocumented immigrants (mostly from Africa) against HBV [49,50,51,52,53,54,55,56,57].

These data suggest considering immigrants from endemic areas at high risk of being carriers of HDV infection, on a par with IVDU, men or women with multiple sexual partners and cohabitants with HBV/HDV-positive family members.

Concluding on this point, we believe that the increasing spread of HDV infection associated with migration flows is becoming a new challenge in the third millennium.

## 4. From HDV Infection to Liver Cirrhosis and HCC, Here Too Something New

HDV can only be transmitted in the presence of a concomitant HBV infection, by coinfection or superinfection [58]. Co-infection is the simultaneous HBV and HDV acute infection occurring in a susceptible individual. HBV/HDV coinfection is responsible for acute hepatitis resembling acute hepatitis B [59], which is frequently more severe than acute hepatitis B and provides an increased risk of acute liver failure [60,61]. As HBV infection develops before HDV infection, HBV/HDV coinfection may cause biphasic peaks in aminotransferases (AST, ALT) serum levels, even weeks apart [60,61]. As HBV is essential for HDV replication, the rate of progression to chronicity is similar to that observed in acute hepatitis B, ranging between 2% and 5% [62,63].

Chronic HBV carriers who acquire HDV infection (HDV superinfection) develop acute hepatitis Delta that progresses to chronicity in nearly three quarters of cases, frequently induces worsening of the pre-existing liver disease and the development of fulminant hepatitis in 7–15% of cases [64]. In the remaining cases HDV replication stops, allowing a less rapid, sometimes indolent course of the disease. In an Italian cohort study, 10% of HBsAg-positive patients with circulating anti-HDV antibodies cleared HBsAg during a mean follow-up period of 4 years, a percentage more than double what happens in patients with HBV mono-infection [65].

HDV chronic infection, detectable by the persistence of antibody to HDV and HDV RNA or HD-Ag in serum for at least 6 months after HDV infection, leads to chronic hepatitis Delta (HDV-CH), a disease more severe than HBV-CH, with higher aminotransferase levels and increased rates of fibrosis progression [66,67]. In addition, patients with HDV-CH are 2-fold more likely to develop and die of hepatic decompensation or HCC than those with HBV mono-infection [63,68,69]. The probability of 20-year survival after the diagnosis of HDV-CH has been estimated at 86%, the persistence of HDV replication being the only factor associated with an increased risk of mortality [63].

HBV/HDV coinfection has been identified as a main factor for the development of HCC in most studies, with a 3-fold increased risk compared to HBV infection [32,63,68,69,70,71,72,73,74]. In a few studies, however, HDV did not appear to significantly increase the risk of developing HCC [32,75]. Understanding this difference would require further investigation, but a different selection of patients by age, HDV genotype (not determined in most studies) and ethnic and environmental aspects is conceivable.

Let us focus now on the most recent data to analyze any differences compared to past. In a recent multicenter study at four hospitals in Spain, 151 (5.2%) out of 2888 HBsAg-positive subjects were anti-HDV positive, of whom 118 had a median follow-up of 8 years. Of these 118, 73% had initially detectable HDV-RNA and 30% liver cirrhosis, most often in HDV-RNA positive patients. Non-cirrhotic patients with initially detectable HDV-RNA were more prone to developing cirrhosis (31% vs. 0%, *p* = 0.002) and/or liver decompensation (28% vs. 3%, *p* = 0.019) than the HDV-RNA-negative ones [76].

A recent nationwide retrospective French study investigated 375 HDV patients with compensated cirrhosis; positive HDV-RNA at the most recent evaluation, an older age, being overweight, total serum bilirubin >17 mmol/L, and low platelet count were all identified as independent factors associated with liver decompensation. In a further analysis, the presence of HDV RNA at the most recent evaluation (HR = 2.14, *p* = 0.01), an older age (HR = 1.08, *p* < 0.001), past alcohol intake (HR = 2.39, *p* = 0.010), prothrombin time < 80% (HR = 4.15, *p* < 0.001), platelet count <100,000/mm^3^ (HR = 2.56, *p* = 0.016) and serum GGT >2-times the normal value (HR = 3.70, *p* = 0.002) were identified as independent factors associated with the development of HCC [77].

The long-term impact of HDV viremia on the outcome was analyzed also in a Swedish nationwide cohort of 337 HBV/HDV co-infected patients, prevalently HDV RNA positive. A significantly increased risk of liver events was seen in patients with HDV-RNA viremia at baseline (HR = 3.82, 95% CI 1.48–9.82), cirrhosis at baseline (HR = 10.26, 95% CI 5.47–19.23) and an older age (HR =1.05, 95% CI 1.03–1.08); the incidence rate per person/year was 2.81% for the HDV- RNA-positive and 0.76% for the HDV-RNA-negative. In the same cohort, a significantly higher risk of HCC development was seen in older patients (HR = 1.08 CI 95%: 1.04–1.13) (*p* < 0.001) and in those with cirrhosis (HR = 3.16 CI 95%: 1.22–11.13) (*p* = 0.02). The risk for HCC development was 0.73% per person/year for HDV-RNA-positive and 0.29% for HDV-RNA-negative patients, a difference however not reaching statistical significance [78].

The influence of the HDV genotype in the evolution of the disease is still under investigation. In a study from Taiwan, 51 patients infected with HDV genotype 1 showed a lower remission rate (15.2% vs. 40.2%; *p* = 0.007) and a more adverse outcomes (cirrhosis, hepatocellular carcinoma, or mortality) (52.2% vs. 25.0%; *p* = 0.005) than 74 patients with HDV genotype 2 [79]. Recent data from Roulot et al. indicate that European patients with HDV genotype 1 and African patients with HDV genotype 5 are at a high risk of developing cirrhosis [77]. In a small British cohort considering 21 African and 9 European patients with HBV/HDV coinfection, those born in Africa were all infected with HDV-5 and showed a better prognosis than those born in Europe, which were mostly infected with HDV-1 [80]. HDV genotype 3 has been linked to outbreaks of severe hepatitis, frequently progressing to acute liver failure and death in the Amazon Basin [28,81,82]. Genotype 4 has been found associated with a mild liver disease [83], but its variant affecting 40 Japanese patients on the Miyako Island has been found to be associated with a more rapid progression to cirrhosis than observed in Taiwan [84]. Further studies on more numerous case series will better highlight the real role of HDV genotypes in influencing the clinical course of chronic hepatitis due to HBV/HCV coinfection.

We certainly did not expect extreme variations in the clinical aspects associated with HDV infection, given the significant volume of studies in this regard produced in past years. The data reported above, however, strongly underline the persistence of HDV viremia as a strong predictor of a poor prognosis; this unfavorable influence is widely confirmed for the evolution to cirrhosis, while a moderate uncertainty persists on its effect on HCC development.

The recent increase in HDV genotypes 5–8 in Western countries is most definitely new. This has already caused and will cause even more changes in the clinical presentation and evolution of HDV-CH. It is therefore important to perform ad hoc studies to acquire rapid clinical information on HDV infection in territories where HDV genotypes 5-8 are spread.

## 5. Therapeutic Attempts for HDV-CH

### 5.1. A Historic Reminder of Treatments with Standard Interferon-α

Standard INF-α was used to treat HDV-CH from 1990 [85,86]. A complete response to this drug, assessed on the persistent normalization of serum alanine aminotransferase values (ALT) and serum HDV RNA clearance, was variable in different studies, but insufficient. In fact, in some studies this treatment obtained the normalization of aminotransferases only while HDV RNA remained detectable [87], and nearly half of the 30–50% of patients with a complete initial response relapsed, an event proportional to the dose and duration of drug administration [88,89].

Treatment of chronic hepatitis D with IFN-α-2a was evaluated by Farci et al. [85] in three groups of 14 HDV infected patients treated for 48 weeks respectively with 9 million IU 3-times a week, or with 3 million IU 3-times a week or left untreated. By the 48th week of treatment, HDV RNA had become undetectable in serum of 71%, 36% and 0% of patients in the high dose, low dose and control cohorts, respectively. A complete response was achieved by 50% of patients in the high-dose IFN group, in 21% of those in the low-dose group and in none of those left untreated. The biochemical response persisted in 50% of patients in the high-dose group up to 12 years, but the effects on viral replication were not sustained. Despite this, the high dose treatment was associated with a reduction of interface hepatitis, lobular necrosis, portal inflammation and stage of fibrosis after an average of 11.5 years. Survival was significantly longer for patients who received the high-dose IFN, compared with those who received the low-dose and with those left untreated; notably, a 2 log10 IU/mL decline in HDV RNA at end of treatment was associated with an increase in survival [90].

The utility of IFN treatment was confirmed in a meta-analysis of 5 studies comparing IFN-α versus no treatment [91]. Virological failure, evaluated at the end of treatment, was observed in 62/92 (67.4%) treated patients and in 71/77 (92.2%) left untreated. The lack of ALT normalization at the end of treatment was observed in 60/92 (65.2%) of treated patients versus 76/77 (98.7%) in the untreated control group. SVR was not achieved by 76/92 (82.6%) of treated patients and by 73/77 (94.8%) of those in the control group. Six months after treatment discontinuation, a high ALT serum value was observed in 81/92 (88.0%) patients treated with IFN compared with 76/77 (98.7%) in the control group. There was no histological improvement in 67/92 (72.8%) patients treated compared with 65/77 (84.4%) in the control group.

As treatment with standard IFN-α obtained unsatisfactory results even when administered in high dosages, in the face of frequent and serious adverse reactions, this drug was replaced by pegylated interferon-α (peg-IFN-α).

### 5.2. Treatment of HDV-CH with peg-IFN-α

Current guidelines from the American Association for the Study of Liver Diseases (AASLD) and the Asia Pacific Association for the Study of the Liver (APASL) suggest the administration of peg-IFN-α for 12 months to subjects with elevated HDV RNA and ALT serum levels [92,93]; similarly, the guidelines of the European Association for the Study of the Liver (EASL) recommend administering peg-IFN-α at least for 48 weeks to HDV-CH patients with compensated liver disease [94]. The HepNet-Greece cohort study observed that INF-based treatment decreases the disease progression in HDV-positive patients (HR = 0.14; 95% CI, 0.02–0.86; *p* = 0.033) [34], and several other authors have identified as an independent predictor of a worse outcome the lack of interferon therapy [68]. However, as for standard IFN-α, the major limitations for peg-INF-α therapy are both the strong contraindication in patients with associated autoimmune diseases and in those with major psychiatric syndromes and the well-known adverse reactions, including flu-like symptoms, myalgias, arthralgias, exacerbation of psychiatric illness, hematologic toxicity and serum aminotransferase values, suggesting an acute exacerbation of liver cell necrosis [95]. In addition, compared with non-cirrhotic patients, the efficacy of peg-INF-α is lower in Child A cirrhotic patients and is considered dangerous for those in the Childs B or C stages, due to fear of disease reactivation and liver failure [25]. Nevertheless, in patients who respond to peg-INF-α therapy, a decrease in HDV-RNA serum levels is usually observed one week after the start of treatment [96]. In addition, either HDV-RNA serum clearance, or a decrease in serum HDV RNA greater than 2 log10 IU/mL, or a decrease in serum HBsAg titers below 1000 IU/mL by the 6th month of treatment are all considered independent predictors of SVR [97,98,99,100].

Two types of peg-IFN-α have been investigated in trials, peg-IFN-α-2a and peg-IFN-α-2b, which appear to have similar efficacy. Among patients treated with Peg-IFN-α-2b (1.5 μg/kg/QW) for one year, 43% were HDV-negative after a median post-treatment follow-up of 16 months (range, 6–42 months) [85], and some other studies reported a SVR rate ranging from 25% to 40% [101,102].

A relapse after treatment discontinuation has been described as frequent during post-treatment follow-up periods. For example, 9 of the 16 patients who cleared serum HDV RNA in the HIDIT-1 trial became HDV-RNA-positive again during a long-term post-treatment follow up [103].

The optimal treatment duration of peg-IFN-α therapy is still undefined and treatment beyond one year is controversial. Karaca et al. reported that more than half of their patients who received peg-IFN-α for 2 years achieved SVR [104]. A recent retrospective investigation showed that the SVR rates observed 2 years after treatment discontinuation increased with the increase in treatment duration, reaching 50% with a 5-year treatment [105].

The 2016 AASLD guidelines recommend 180 μg peg-IFN-α, one injection QW for 48 weeks, for treating adult HDV-CH patients [106], since administering increasing doses up to 360 μg QW for up to 5 years or extending peg-IFN-α-2b therapy to 72–102 weeks does not increase the SVR rate over 35% [97,99].

Noteworthy, HDV RNA serum levels detected at the 24th week of treatment may predict who, among patients treated with peg-IFN-α for 48 weeks, is likely to test negative 24 weeks after the end of treatment [97].

### 5.3. Treatments with HBV Nucleo(s)tide Analogues, Given Alone or in Combination with peg-IFN

Administered alone, these HBV nucleo(s)tide analogues have provided no benefit to HDV-CH patients [107,108]. The efficacy of oral lamivudine on HDV RNA, ALT, liver histology and HBsAg seroconversion was evaluated in a multicenter randomized-controlled study. Thirty-one patients with HDV-CH were randomized to receive either lamivudine 100 mg daily or placebo for 52 weeks and, subsequently, those treated with lamivudine received the same treatment for an additional 52 weeks and were observed post-treatment for 16 weeks. No patient became HDV-RNA-negative after 52 weeks of treatment and 3 were found to be negative after 104 weeks, of whom 2 remained so at week 120 [109].

Due to the limited efficacy of interferon on HDV-CH, several studies evaluated the efficacy of peg-IFN-α combined with HBV nucleo(s)tide analogues [110,111]: lamivudine [108,112], famciclovir [113], adefovir [101], tenofovir [114] and entecavir [107]. HBV nucleo(s)tide analogues, however, did not provide additional advantage over peg-IFN-α alone [101,114,115]. This was found by the HIDIT-I randomized trial that enrolled 90 patients, 30 treated with adefovir (10 mg oral daily), 29 with peg-IFN-α-2a (180 μg/QW) and 31 with adefovir + peg-IFN-α-2a for 48 weeks; patients were followed up untreated for an additional 24 weeks. At the end of follow up, HDV RNA was negative in 0%, 24% and 23% of patients in the ADV, peg-IFN and combination group, respectively [101]. The multivariate analyses of the HIDIT-I study revealed that the level of HDV RNA at treatment week 24 is predictive of HDV RNA serum clearance, as detected 24 weeks after the end of treatment [97]. Similar data were obtained for tenofovir + Peg-IFN in the HIDIT-2 study, including two parallel, multicenter, double-blind, randomized, controlled trials evaluating the efficacy of peg-IFN-α-2a (180 μg/QW) given for 96 weeks in combination with tenofovir disoproxil fumarate (TDF), 300 mg daily to 59 patients, versus Peg-IFN-α-2a + placebo given for 96 weeks to 61 patients [116]. The addition of TDF did not improve the rate of HDV RNA clearance at the end of treatment, nor did it reduce the viral recurrence after treatment discontinuation. Nevertheless, the 96-week peg-IFN-α-2a treatment was well tolerated by most patients and provided a reduction in the liver fibrosis scores.

Also peg-IFN-α plus ribavirin or adefovir combination therapies did not increase the rates of HDV RNA serum clearance [101,102].

Based on these and some other landmark studies, current guidelines suggest considering an HBV nucleo(s)tide analogue in addition to peg-IFN-α only for the few HDV-CH patients with ongoing HBV DNA replication [92,94].

### 5.4. Therapeutic Agents Interfering with the HDV Life Cycle: Bulevirtide, Lonafarnib and Nucleic Acid Polymers

Three molecules are candidates as new therapeutic agents against HDV infection: bulevirtide, lonafarnib and nucleic acid polymers (NAPs) (Figure 2).

Sodium taurocholate cotransporter polypeptide (hNTCP) mediates the entry of HBV and HDV into hepatocytes, acting as a specific receptor [117]; therefore, hNCPT inhibitors may be used as therapeutic agents. The 48 amino acid myristoyl peptide of the L-HBsAg protein inhibits the entry of HBV and HDV into hepatocytes [118] through a competitive binding with hNTCP that hinders the attachment of L-HBsAg. Bulevirtide is a 47 amino acid peptide with an N-terminal myristolic moiety and a C-terminal carboxamide that, like the 48 amino acid myristoyl peptide of the L-HBsAg protein, inhibits the binding of HBsAg to NTCP [117]. In a pilot randomized, open-label study of phase 1b/2a, the Authors enrolled 24 patients with HDV-CH to evaluate the effect of bulevirtide, either administered alone or in combination with peg-IFN-α-2a, in comparison with peg-IFNα-2a alone [119]. More precisely, 8 patients received 2 mg bulevirtide daily subcutaneously for 24 weeks followed by peg-IFN-α-2a QW for 48 weeks (bulevirtide group), while 8 other patients received 2 mg bulevirtide daily in combination with peg-IFNα-2a QW for 24 weeks followed by 24 weeks of peg-IFNα-2a QW alone (bulevirtide-peg-IFN group), and the remaining 8 were treated with peg-IFN-α-2a QW alone (peg-IFN group) [119]. A decrease in HBsAg serum values was not achieved in any group, but the mean HDV RNA declines at the 24th week of treatment were −1.67, −2.6 and −2.2 log in bulevirtide, bulevirtide-peg-IFN and peg-IFN groups, respectively. It is noteworthy that HDV RNA was below the limit of detection in 5 patients in the bulevirtide-peg-IFN group and in 2 in both bulevirtide and peg-INF groups. At week 48, after the second 24-weeks peg-IFN-α-2a course, no additional benefit occurred in any group [119]. The Authors concluded that each antiviral drug has an activity against HDV infection, enhanced by their use in combination [119].

In 2018, Wedemeyer et al. enrolled 120 patients with HDV-CH and treated them for 24 months in 4 arms. The first arm received TDF 245 mg/day and bulevirtide (Myr B) 2 mg/day subcutaneously (group A), the second one received TDF 245 mg/day and Myr B 5 mg/day (group B), the third one received TDF 245 mg/day and Myr B 10 mg/day (group C) and the last one received TDF 245 mg/day alone (group D) [120]. At the end of treatment, serum HDV RNA was detected to be negative or reduced by at least 2 log in 46.4%, 46.8%, 76.6% and 3.3% of patients in groups A, B, C or D, respectively. The mean HDV RNA serum values declined by −1.75 log, −1.60 log, −2.70 log and −0.18 log IU/mL in the A, B, C and D groups, respectively [120]. ALT normalization was observed in 42.8%, 50%, 40% and 6.6% of patients in these four groups, respectively [121]. Unfortunately, at the end of a 12-week post-treatment follow-up, a virological relapse associated with an elevation in serum ALT had occurred in 60%, 80% and 83% of patients in groups A, B and C, respectively [120].

In 2019, Wedemeyer et al. randomized 60 patients with HBV/HDV coinfection in 4 arms, all of which were treated for 48 weeks and followed up for 24 weeks post treatment [122]. Patients enrolled in arm A received peg-IFNα-2a QW alone, those in arm B 2 mg received Myr B/day and peg-IFNα-2a QW, those in arm C received 5 mg Myr B/day and peg-IFN-α-2a QW and those in arm D received 2 mg Myr B/day alone [122]. Undetectable serum HDV RNA at week 72, chosen by the Authors as the primary endpoint, was achieved by 0%, 53%, 26% and 0% of patients in the four arms, respectively; the median serum HDV RNA log reduction was −0.26 in arm A, −4.04 in arm B, −1.48 in arm C and −1.01 in arm D [122].

More recently, 30 HBeAg-negative patients with chronic HBV/HDV co-infection were randomized in 2 arms and treated for 48 weeks with either 10 mg bulevirtide/day plus peg-IFN-α-2a QW or 5 mg of bulevirtide given twice daily + TDF 245 mg/day [123]. At week 24, the serum HDV RNA level had declined from baseline by −4.84 log10 IU/mL in the Myr B/peg-IFN-α-2a arm and by −2.80 log10 IU/mL in the Myr B/TDF arm; HDV had become undetectable in 60% and 21% of patients, respectively, suggesting a better efficacy of the combination of Myr B + peg-IFN-α-2a [124]. Observing these patients over time would show the percentage of patients who will have eradicated HDV infection. The tolerability to bulevirtide was considered to be good by the Authors, since after 24 weeks of treatment, only 11 adverse reactions had occurred, mostly related to an increase in bile salts serum levels [123].

Ezetimibe is a cholesterol-lowering drug and an inhibitor of NTCP that was evaluated in a proof-of-concept phase 2 trial in 44 HDV-CH patients [125]. This drug, given at a 10 mg/die dose for 12 weeks, showed only a slight activity in reducing HDV RNA levels and serum aminotransferases, which was insufficient for being used in monotherapy [126].

Prenylation is a host cellular process inside hepatocytes that promotes membrane association of proteins and mediates the interaction between proteins [121]. Prenylation makes HD-Ag more lipophilic and promotes its interaction with HBsAg, a key point for the HDV particle formation; moreover, being a host process, a high barrier to resistance is expected [121]. Lonafarnib is an agent that inhibits the addition of farnesyl prenyl-lipid group to HD-Ag, thereby blocking the interaction with HBsAg and inhibiting the secretion process. In 2015, a phase 2a proof-of-concept, randomized, controlled trial investigated the safety of lonafarnib in patients with HDV-CH and its effectiveness in reducing HDV RNA serum values after 28 days of treatment [121]. Fourteen patients were randomized into 2 different dose groups: group 1, of 8 patients, treated with lonafarnib 100 mg twice daily and group 2 of 6 patients treated with 200 mg lonafarnib twice daily [121]. At the 28th day of treatment, the mean HDV RNA decline from the baseline was −0.73 log IU/mL in group 1 (95% CI 0.17–1.31; *p* = 0.03) and −1.54 log IU/mL in group 2 (1.21–1.93; *p* < 0.0001). Lonafarnib serum concentrations correlated with HDV RNA change (r2 = 0.78, *p* < 0.0001) [121]. After treatment discontinuation, HDV RNA levels returned to baseline within 4 weeks in all treated patients [121]. No serious adverse events occurred in either treatment groups, but all the patients included in group 2 experienced nausea, diarrhea, abdominal bloating and weight loss; 83% of them developed anorexia and 50% vomiting [121]. These data induced the research group to identify pharmacological combinations with other drugs to improve the clinical efficacy and limit the adverse events.

The study LOWR HDV-1, a single center phase 2 pilot study, investigated the combination of different dosages of lonafarnib in combination alternatively with ritonavir or peg-IFN-α-2a for 8 or 12 weeks [127]. Twenty-one HDV RNA positive patients were enrolled, 3 in each of 7 different groups; Group 1 was treated with lonafarnib 200 mg BID in monotherapy for 12 weeks, group 2 with a time-increasing dosage of lonafarnib (200 mg BID for 12 weeks and 300 mg BID for a subsequent 12 weeks), group 3 with lonafarnib 100 mg TID for 8 weeks; group 4 with lonafarnib 100 mg BID plus ritonavir (RTV) 100 mg QD for 8 weeks; group 5 with lonafarnib 100 mg BID plus peg-IFNα-2a QW for 8 weeks; group 6 with lonafarnib 200 mg BID plus peg-IFNα-2a QW for 8 weeks, group 7 with lonafarnib 300 mg BID plus peg-IFNα-2a QW for 8 weeks [114]. After 4 weeks lonafarnib in combination or in monotherapy induced a decline in HDV RNA load in treated patients [127]. Lonafarnib in combination with RTV or with peg-IFNα-2a induced the highest decline in the HDV RNA viral load (−3.2 and −3.0 log10, respectively) [127]. The most often observed adverse events were lonafarnib-dependent gastrointestinal events (nausea, vomiting, diarrhea) and weight loss [127]. These data led the authors of the LOWR HDV-1 study to start a new therapeutic attempt with the LOWR HDV-2 study; 48 patients with chronic HDV infection were enrolled and divided into three groups respectively receiving a high dose lonafarnib ≥ 75 mg BID + RTV 100 mg BID (15 patients treated for 12 weeks), or a low dose lonafarnib (25 or 50 mg BID) + RTV 100 mg BID (20 patients treated for 24 weeks), or a low dose lonafarnib (25 or 50 mg BID) + RTV 100 mg BID + Peg-IFN-α QW (13 patients treated for 24 weeks) [128]. The administration of lonafarnib 25 mg BID + RTV + Peg-IFN-α achieved the highest mean log decline (−5.57 log10 U/mL), suggesting a synergistic activity for the lonafarnib + RTV+ Peg-IFN-α combination [124].

The LOWR-HDV-4 [127] study is a dose-escalation study performed to investigate whether a rapid stepwise increase in the lonafarnib dosage from 50 mg to 100 mg BID (associated with ritonavir 100 mg BID fixed dosage and HBV nucleo(s)tide analogue) might increase the number of patients achieving higher lonafarnib doses [129]. If well tolerated, lonafarnib was increased to 75 mg BID after at least 4 weeks, and then to 100 mg BID after at least 2 weeks from the previous escalation [125]. Fifteen patients were enrolled, of whom lonafarnib dose-escalation from 50 to 100 mg BID was possible in 10 patients (66%), indicating the possibility of individualized treatment strategies [125].

An open-label Phase 2a study was performed to evaluate the safety and the antiviral effect of lonafarnib/RTV + peg-IFN lambda-1a given for 24 weeks to 26 HDV-CH patients [129]. At the end of treatment, the median HDV RNA decline from baseline was −3.4 log10 (*p* < 0.0001), with 7 patients become HDV RNA negative and 3 with serum HDV RNA not quantifiable. Adverse events were mild or moderate in most of cases and prevalently related to the gastro-intestinal system [129].

NAPs are amphipathic single stranded phosphorothioated oligonucleotide broad-spectrum antiviral agents. Their antiviral activity is based on the interaction with large amphipathic protein domains, which is necessary for viral replication [130]. The NAPs REP2139 clears circulating HBsAg by blocking the release of both HBV and HDV sub-viral particles [128].

In open-label, non-randomized, proof-of-concept Phase 2 study, Bazinet et al. demonstrated the good tolerability and safety of REP2139 in combination with peg-IFN-α-2a [131]. These Authors treated 12 HDV-CH patients with REP2139 500 ng intravenous QW for 15 weeks, followed by a 15-week treatment with REP2139 QW + peg-IFN-α-2a QW and finally by a 33-week treatment with peg-IFN-α-2a QW [131]. The combination therapy of REP2139 + peg-IFN-α-2a established functional control of HBV and HDV coinfection [131]. The most common adverse events observed during REP2139 monotherapy were pyrexia, chills, conjunctival hyperemia, headache and asthenia; 4 serious adverse events were linked to peg-IFN-α-2a. In terms of efficacy, 11 patients became HDV-RNA-negative during treatment, 9 of whom remained so at the end of treatment and 7 also after 1-year of post-treatment follow-up [131].

Concluding on this point, bulevirtide and lonafarnib are antiviral agents with good anti-HDV activity, in an advanced phase of investigation. However, the high frequency of relapses and adverse reactions makes it essential to be cautious in the interpretation of the available data. The association of these antiviral agents with other drugs, a solution recently undertaken, makes it seem advisable to improve their therapeutic effect and temper the adverse reactions. To date little can be said about NAPs, as the available information comes from a single phase 2 study, which however shows the efficacy of REP2139 in clearing serum HDV RNA in half of the treated patients up to one year after drug discontinuation. Even for this drug, the numerous adverse events represent a serious limit, which hopefully might be overcome by combination therapies.

## 6. Discussion

Several areas of the complex HDV theme have been explored in recent times. The interaction between HDV and HBV has been clarified in its biochemical and molecular aspects and this has made it possible to identify agents active in interfering in the HDV life cycle.

The intensification of migratory flows from areas at high HDV endemicity to Western countries have partially modified the epidemiology of HDV infection of these geographical areas, especially with the introduction of new genotypes, whose clinical impact is not yet known, but certainly deserves attention.

## 7. Conclusions

Old and new data indicate that a prolonged ongoing of HDV replication is a strong independent predictor of development of cirrhosis and HCC, highlighting the need for drugs able to eliminate viral synthesis.

Ideally, an effective therapy should also result in the elimination of HBV infection with the consequent impossibility of HDV to survive the elimination of its helper virus. In the absence of such therapy, many attempts have been made to find a valid response to the poor prognosis often associated with chronic HDV infection. Most of these attempts have been based on the prolonged administration of high dose standard IFN-α or peg-IFN-α. This treatment leads to a normalization of aminotransferase serum levels and HDV RNA undetectability in 20–50% of patients during treatment. This effect, however, is transitory in about half of treated cases. Furthermore, the addition of HBV nucleo(s)tide analogues to peg-IFN-α does not improve the rate of patients with SVR, most probably because the helper effect of HBV cccDNA is sufficient for HDV to maintain its replicative activity and its harmful capacity. The inadequacy of these therapeutic attempts is even more evident if compared with their poor tolerability and to the frequent serious adverse reactions entailed.

The improved knowledge on the HDV life cycle has provided the basis for the development of agents directly acting towards some essential points of the HDV life cycle. These drugs appear destined to enhance the therapeutic response but, according to the suggestion of the current AASLD guideline, they should only be administered in referral specialized centers at present.

We hope that this narrative review will be useful for doctors and researchers in liver disease and especially for trainees and young specialists in infectious diseases, gastroenterology and internal medicine.

## Data Availability

Data sharing not applicable—no new data generated. Data sharing is not applicable to this article as no new data were created or analyzed in this study.
